# Cerebrospinal fluid analysis in Guillain–Barré syndrome: value of albumin quotients

**DOI:** 10.1007/s00415-021-10479-9

**Published:** 2021-03-02

**Authors:** Jakob Rath, Gudrun Zulehner, Bernadette Schober, Anna Grisold, Martin Krenn, Hakan Cetin, Fritz Zimprich

**Affiliations:** Department of Neurology, Medical University of Vienna, Währinger Gürtel 18-20, 1090 Vienna, Austria

**Keywords:** Cerebrospinal fluid, Lumbar puncture, Guillain–Barré syndrome, Albuminocytologic dissociation, Protein

## Abstract

**Background:**

Albuminocytologic dissociation in cerebrospinal fluid (CSF) is a diagnostic hallmark of Guillain–Barré syndrome (GBS). Compared to CSF total protein (TP), the CSF/serum albumin quotient (Qalb) has the advantage of method-independent reference ranges. Whether the diagnostic yield differs between Qalb and CSF-TP is currently unknown.

**Methods:**

We retrospectively analyzed the diagnostic yield (i.e., a value above the URL indicating blood–nerve barrier dysfunction) of Qalb and CSF-TP levels in patients with GBS. We evaluated two different equations (Reiber’s and Hegen’s) for age-adjusted URLs of Qalb and compared results to CSF-TP using the standard URL of 0.45 g/L as well as age-adjusted URLs (by decade of age). Additionally, multivariable logistic regression analysis was used to assess the effect of clinical factors on the diagnostic yield.

**Results:**

We analyzed 110 patients [62% males; median age 48 (IQR 35–58)] with sensorimotor (68), motor (16), sensory (12) and localized (8) GBS as well as Miller Fisher syndrome (6).

Qalb and CSF-TP were highly correlated (*r* = 0.95, *p* < 0.001). The diagnostic yield of Qalb was 65% with Reiber’s and 47% with Hegen’s age-adjusted URLs compared to 66% with the fixed CSF-TP URL of 0.45 g/L and 49% with age-adjusted CSF-TP URLs. A longer duration from clinical onset to lumbar puncture was associated with a higher diagnostic yield.

**Conclusion:**

Qalb strongly correlates with CSF-TP in patients with GBS with a similar diagnostic yield for the detection of a blood–nerve barrier dysfunction. However, the diagnostic yield of both values is lower when using more recent age-adjusted URLs and at earlier timepoints.

## Background

The Guillain–Barré syndrome (GBS) is an acute immune-mediated neuropathy with a range of clinical subtypes [1]. Diagnostic criteria, such as the Brighton criteria [2] for classical GBS, focus on the typical clinical presentation in conjunction with electrophysiological results as well as the presence of an albuminocytologic dissociation in the cerebrospinal fluid (CSF). The latter has been a hallmark of GBS since its first description, as it reflects disruption of the blood–CSF barrier due to nerve inflammation [3, 4]. However, inflammation in GBS is variable between patients and elevated protein levels are not found in all cases. Albuminocytologic dissociation was reported to range between 44 and 81% and depends on the timing of lumbar puncture (LP) with lower numbers early in the disease course [5–10]. Additionally, patients with less common, localized variants or with Miller Fisher syndrome have lower rates of albuminocytologic dissociation [6–8, 10].

Moreover, protein levels in the CSF have been shown to be age-dependent [11] and sensitivity of total protein (TP) for the detection of albuminocytologic dissociation is further reduced when age-adjusted values are used [9]. Whether using CSF/serum albumin quotients (Qalb) increases the diagnostic yield in comparison to CSF-TP level is currently unknown. The benefits of Qalb are the method-independent reference range allowing comparison between laboratories, its independence from intrathecal protein synthesis and the correction for plasma albumin concentration [3].

In this study, we retrospectively analyzed CSF results in a cohort of clinically well-characterized patients with different GBS subtypes.

The aims of the study were to investigate

- whether using Qalb increases diagnostic yield (i.e., a value above the URL indicating blood–nerve barrier dysfunction) in comparison to CSF-TP
- by comparing two different equations for age-adjusted upper reference limits (URLs) for Qalb and
- to evaluate clinical factors that may have an effect on the diagnostic yield of Qalb and CSF-TP.

## Methods

### Patients

We retrospectively examined clinical data of adult patients with GBS who were treated at the Department of Neurology of the Medical University of Vienna between January 2000 and December 2019. The study was approved by the ethics committee of the Medical University of Vienna (EK Nr. 1927/2016). The requirement to obtain patient consent was waived for this retrospective study.

### Patient data

We extracted clinical data from patient charts and grouped patients clinically into sensorimotor or pure motor classical GBS, pure sensory GBS and localized variants (pharyngeal–cervical–brachial variants and bilateral facial palsy with paresthesia) as well as Miller Fisher syndrome (including incomplete forms; patients with limb weakness were classified as classical GBS). We retrospectively calculated the Medical Research Council (MRC) sum score at admission from patient charts. The score ranges from 0 (indicating complete paralysis) to 60 (normal strength) points. Nerve conduction studies were analyzed according to Rajabally’s criteria [12]. Ganglioside antibodies were measured in sera of patients using enzyme-linked immunosorbent assays (ELISAs).

### CSF samples

All CSF samples were obtained during routine evaluation in patients with clinically confirmed GBS and the analysis was carried out in the central laboratory of the Medical University of Vienna at the same day. We analyzed Qalb (Alb_CSF_/Alb_serum_) with age-adjusted URL (Qalb × 10^–3^) as previously suggested by Reiber [13]:

$$\mathrm{Qalb}=4+\frac{\mathrm{Age}}{15}.$$

Additionally, we calculated reference values for the 95th percentile of Qalb using the more recent age-adjusted equation by Hegen and colleagues (age × 0.0435 + 7.9249) in the suggested rounded version [14]:

$$\mathrm{Qalb}=\frac{\mathrm{Age}}{25}+8.$$

CSF-TP levels were analyzed with a standard fixed cut-off of 0.45 g/L and with age-adjusted URL (97.5th percentile) for each decade of age based on the large cohort of McCudden et al. [15] and described in Bourque et al. [11], which are roughly similar to other recent suggested reference intervals [14, 16]. Age-adjusted URLs by decade are: 18–29 years: 0.49 g/L; 30–39 years: 0.55 g/L; 40–49 years: 0.58 g/L; 50–59 years: 0.6 g/L; 60–69 years: 0.64 g/L; 70–79 years: 0.68 g/L. For the single patient above 79 years, the reference range of patients aged 70–79 was used given the limited data in this age group.

### Statistical analysis

SPSS 26 software package (IBM Corp. Released 2019. IBM SPSS Statistics for Macintosh, Version 26.0. Armonk, NY: IBM Corp), R version 4.02 (R Core Team, 2020. R: A language and environment for statistical computing. R Foundation for Statistical Computing, Vienna, Austria) and R Studio version 1.3.959 (RStudio Team, 2020. RStudio: Integrated Development for R. RStudio, PBC, Boston, MA) were used for statistical analysis.

Median values between GBS subtypes were compared with SPSS’s independent-samples median test. Pearson correlation coefficients were calculated between Qalb, CSF-TP and time from symptom onset to LP after removal of outliers (values above the third quartile plus 2.2 times the IQR). Correlation coefficients were interpreted as low (*r* ≤ 0.35), moderate (*r* 0.36—0.67) or high (*r* > 0.68) correlation [17]. Dichotomous outcomes of Qalb and CSF-TP (i.e., value above the URL) were analyzed using multivariate logistic regression with sex and GBS subtype as categorical covariates and age at onset, MRC sum score at admission as well as time from symptom onset to LP as continuous covariates including all two-way interactions. *p* ≤ 0.05 was considered statistically significant.

## Results

We retrospectively investigated 129 adult patients with the diagnosis of an acute immune-mediated neuropathy between 2000 and 2019. We excluded 3 patients because no lumbar puncture (LP) was performed in-house, 1 patient because no LP was available within the first 30 days after clinical onset, 4 patients because of macroscopic visible artificial blood contamination due to traumatic LP and 11 patients because albumin was not measured in CSF. The remaining 110 patients [62% males, median age 48 (IQR 35–58)] were subsequently analyzed. Patients were clinically classified as sensorimotor (68), motor (16), sensory (12) and localized (8) GBS as well as Miller Fisher syndrome (6). The MRC sum score at baseline was 54 (IQR 46–60). Baseline characteristics are shown in Table 1.

**Table 1:** Clinical characteristics

	*N* = 110
Sex	68 (62%) males, 42 (38%) females
Median age (IQR; range)	48 (35–58; 20–84)
GBS subtype	
Classic sensorimotor	62% (68/110)
Classic pure motor	15% (16/110)
Pure sensory	11% (12/110)
Miller Fisher syndrome	5% (6/110)
Localized variant	7% (8/110)
Preceding infection	
Gastrointestinal	32% (35/110)
Respiratory	16% (17/110)
Other	14% (15/110)
NCS (Rajabally’s criteria)	
Demyelinating	25% (27/107)
Axonal	23% (25/107)
Equivocal	40% (43/107)
Normal	11% (12/107)
IgG antibodies against gangliosides	
GM 1	14% (9/64)
GQ1b	11% (6/56)*
Median creatinine level (IQR)	0.88 mg/dL (0.74–1-03)
Median TSH (IQR)	1.66 µlU/mL (1.24–2.35)
Median MRC sum score at admission (IQR)	54 (46–60)
GBS disability scale at nadir	
1	18% (20/110)
2	40% (44/110)
3	10% (11/110)
4	22% (24/110)
5	10% (11/110)
6	0

Clinical characteristics of the 110 patients with GBS included in the analysis. The GBS disability scale is shown for clinical severity at disease nadir and ranges from 0 to 6 with higher scores indicating more severe disease at nadir. The MRC sum score was calculated at admission and ranges from 0 to 60 with lower values indicating more severe paresis

IQR denotes interquartile range, MRC Medical Research Council and NCS nerve conduction study. * Anti-GQ1b antibodies were observed in four patients with MFS, 1 patient with MFS/ GBS overlap and 1 patient with a regional variant

### CSF results

The median time from clinical onset to LP was 5 days (IQR 3–10, range 0–30 days).

All patients had cell counts below 50 cells/µL (median of 2 cells/µL; IQR 1–4, range 0–28) as required by the Brighton criteria [2].

#### Albumin quotients and CSF total protein levels

**Fig. 1:**
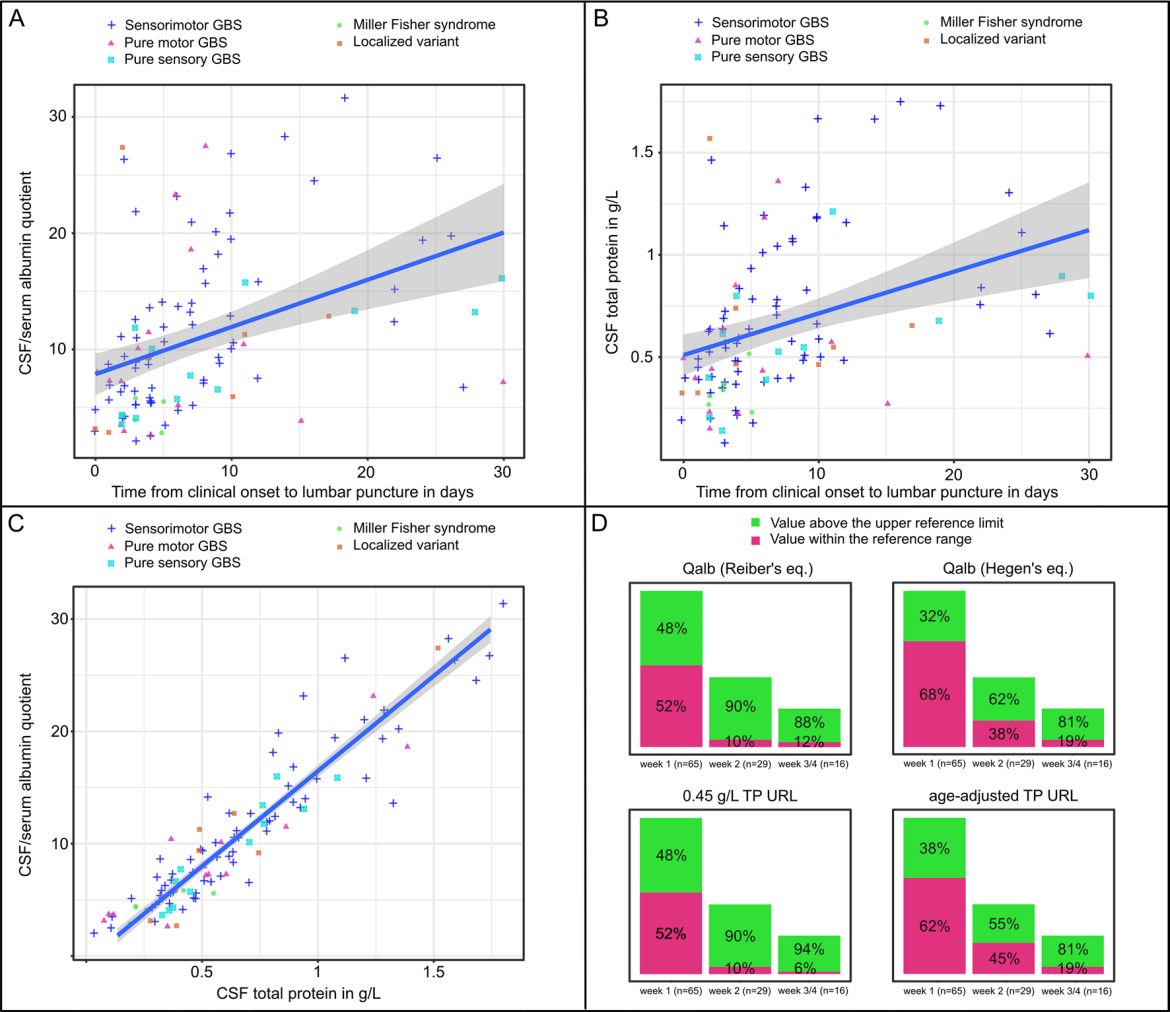
Albumin quotient and total protein in CSF. Scatterplots in A-C show results of CSF evaluations of each individual patient with removed outliers (values above the third quartile + 2.2 × IQR). Fitted regression lines with 95% confidence interval are displayed and GBS subtypes are depicted as differently colored shapes. **a** Scatter plot of CSF/serum albumin quotient and time from symptom onset to lumbar puncture in days. **b** Scatter plot of CSF total protein and time from symptom onset to lumbar puncture in days. **c** Scatter plot of CSF/serum albumin quotient and CSF total protein. **d** Bar plots show relative percentages of patients with values above the upper reference limits according to time of lumbar puncture from symptom onset grouped by weeks. The upper half shows results for CSF/serum albumin quotients (Qalb) with reference limits according to Reiber’s equation [13] and Hegen’s equation [14]. The lower half shows results for CSF total protein (TP) using a upper reference limit of 0.45 g/L and age-adjusted reference limits (by decade of age) [15]

The median Qalb was 9.3 (IQR 5.6–14.3; range 2.1–118.1) and median total protein levels were 0.58 g/L (IQR 0.39–0.87; range 0.14–4.78) with a high linear correlation between the two variables (*r* = 0.95, 95% CI 0.93–0.97; *p* < 0.001; Fig. 1c). A moderate correlation was found between time from symptom onset to LP and both Qalb (*r* = 0.41, 95% CI 0.23–0.55; *p* < 0.001) and CSF-TP (*r* = 0.37, 95% CI 0.19–0.52; *p* < 0.001). Figure 1a shows a scatter plot with fitted regression lines and 95% confidence intervals of individual Qalb values versus time from symptom onset to LP according to GBS subgroups and Fig. 1b provides the same for CSF-TP.

There was no statistically significant difference between GBS subgroups for Qalb (*p* = 0.75) and CSF-TP values (*p* = 0.054). Table 2 shows results of albumin quotients and total protein levels according to GBS subgroups.

**Table 2:** Diagnostic yield of Qalb and CSF-TP

	All patients (*n* = 110)	SM-GBS (*n* = 68)	M-GBS (*n* = 16)	S-GBS (*n* = 12)	MFS (*n* = 6)	Loc-GBS (*n* = 8)
Median time from onset to LP (IQR)	5 (3–10)	5 (3–9.75)	5 (2–10.25)	6.5 (3–17)	3 (2–5)	4 (1.25–10.75)
Median cell count (cells/µL) (IQR)	2 (1–4)	3 (2–4)	2 (1–5)	2 (1–5)	2 (1–3)	2 (1–6)
Median Qalb (IQR)	9.3 (5.6–14.3)	10.3 (6.3–16.6)	7.7 (4.2–16.8)	8.9 (4.7–13.3)	4.3 (3.7–5.7)	9.3 (3.9–12.4)
Above age-adjusted URL (Reiber’s equation [1])	65%	72%	56%	58%	0%	75%
Above age-adjusted URL (Hegen’s equation [2])	47%	50%	44%	50%	0%	63%
Median CSF-TP g/L (IQR)	0.58 (0.39–0.87)	0.61 (0.44–0.97)	0.48 (0.30–1.09)	0.61 (0.40–0.83)	0.32 (0.27–0.38)	0.59 (0.29–0.71)
Above 0.45 g/L	66%	72%	63%	67%	0%	63%
Above age-adjusted limit (by decade of age [3, 4])	49%	54%	38%	50%	0%	63%

Qalb and CSF-TP levels are shown as well as the rate of patients with values above the upper reference limits. CSF cerebral spinal fluid, GBS Guillain–Barre syndrome, IQR interquartile range, LP lumbar puncture, M motor, MFS Miller Fisher syndrome, loc localized, SM sensorimotor, CSF-TP CSF total protein, URL upper reference limit

#### Diagnostic yield

The diagnostic yield (i.e., a value above the URL) was 65% (71/110 patients) with Reiber’s equation for age-adjusted URLs of Qalb and 47% (52/110) with Hegen’s equation. For CSF-TP, the diagnostic yield was 66% (72/110) using the fixed URL of 0.45 g/L and 49% (51/110) using the age-adjusted URLs. 45% (49/110) of patients had CSF-TP and Qalb values above all 4 analyzed URLs and 32% (35/110) had consistently values within the normal range. Table 2 shows results according to GBS subgroups.

The multivariate logistic regression model showed that only time from symptom onset to LP had a significant adjusted effect on the diagnostic yield. Specifically, the estimated odds ratios (referring to an increase of the variable *time from symptom onset to LP* per day) for a value above the URL (vs. a value within the normal range) were 1.22 with Reiber’s equation (95% CI 1.07–1.38, *p* = 0.003), 1.11 with Hegen’s equation (95% CI 1.03–1.20, *p* = 0.003), 1.63 for a CSF-TP URL of 0.45 g/L (95% CI 1.17–2.28, *p* = 0.004) and 1.09 for age-adjusted CSF-TP URLs (95% CI 1.02–1.17, *p* = 0.014). No significant adjusted effects were found for age at onset, sex, MRC sum score at admission and GBS subgroups. Of note, all six patients with MFS had Qalb and CSF-TP values within the normal range but all had their LP done within the first 5 days after clinical onset.

## Discussion

In this study, we retrospectively investigated the diagnostic yield (i.e., a value above the URL indicating blood-nerve barrier dysfunction) of Qalb compared to CSF-TP in patients with GBS. The main finding was that Qalb highly correlated with CSF-TP and had a similar diagnostic yield. The latter was, however, lower for both values at earlier timepoints and when using more recent age-adjusted URLs.

To our knowledge, no previous study investigated Qalb in patients with GBS in comparison to CSF-TP. With regard to the detection of a blood–nerve barrier dysfunction in patients with GBS, we found no clear superiority of Qalb compared to CSF-TP as demonstrated by the high number of patients with concordant findings regardless of the used URL (77%). Nevertheless, given the high correlation of Qalb and CSF-TP, it might be preferable to use Qalb in scientific settings—e.g., in multicenter studies—due to its laboratory-independent URLs that facilitate pooling of data, respectively, comparison between sites [3].

The diagnostic yield of Qalb and TP was lower with more recently suggested age-adjusted URLs. For CSF-TP, this has already been recently shown in a large cohort of patients with classical sensorimotor GBS in a Canadian study, where age-adjusted reference limits led to a markedly lower proportion of patients with albuminocytologic dissociation compared to the conventional fixed cut-off of 0.45 g/L, especially in the first week after clinical onset [9]. While age-adjusted URLs [14–16] will lead to increased specificity, the reduced sensitivity for the detection of an albuminocytologic dissociation in GBS needs to be taken into account in clinical practice.

As expected, we found that the time from clinical onset to LP correlated, albeit moderately, with CSF-TP and Qalb. A value above the URL in the first week was found in only a third to a half of patients depending on the measure and URL. Beyond 2 weeks, the vast majority of patients had elevated values, which is in line with other reports [5, 9, 18].

Qalb and CSF-TP values did not differ significantly between GBS subgroups, but levels in pure motor GBS were numerically lower compared to sensorimotor GBS. Indeed, this was also reported in a recent study that showed higher CSF-TP levels in primary demyelinating compared to axonal subtypes or MFS [10]. The interpretation of lower levels of Qalb and CSF-TP in MFS patients in our study is limited by the low number of patients, all of which had LP within the first 5 days; however, our median CSF-TP are in line with the reported levels of CSF-TP in the first week in an Asian cohort of MFS patients [8]. Interestingly, patients with pure sensory GBS and localized variants had roughly similar Qalb and CSF-TP levels as those with sensorimotor GBS but the small samples again limited the interpretation; furthermore, a selection bias is likely, since patients with isolated clinical findings are more likely diagnosed with GBS variants in the presence of an albuminocytologic dissociation.

Limitations of this study are the retrospective nature and non-standardized CSF collection.

Furthermore, no information was available on which proportion of the CSF was used for analysis which could have influenced CSF measurements since a previous study showed that protein concentrations decrease between the first (0–4th) milliliters and the last (21st–24th) milliliters [19]. However, standard CSF collection practice at our institution is to take approximately 10–12 ml of CSF partitioned into 4–5 sterile tubes and analysis is usually carried out using one of the first 3 tubes. Moreover, patient positioning during the LP might have influenced protein but could not be considered due to the retrospective nature of this study [20–22]. Finally, we were not able not evaluate performance measures such as specificity or ROC curves of CSF-TP or Qalb because of the absence of a control group (e.g., GBS mimics).

In conclusion, we found that Qalb correlates well with CSF-TP in patients with GBS and has a similar diagnostic yield regarding the detection of a blood-nerve barrier dysfunction. However, the diagnostic yield for both values is lower at earlier timepoints and when using more recent age-adjusted URLs.

## Data Availability

Data can be made available from the corresponding author upon reasonable request and after approval from the ethics review board at the Medical University of Vienna.

## Abbreviations

CI: Confidence interval
CSF: Cerebrospinal fluid
CSF-TP: CSF total protein
GBS: Guillain–Barré syndrome
IQR: Interquartile range
MFS: Miller Fisher syndrome
MRC: Medical Research Council
OR: Odds ratio
Qalb: CSF/serum albumin quotient
URL(s): Upper reference limit (s)
